# Supplementary material for: Transportome remodeling of a symbiotic microalga inside a planktonic host
Source: ISME J. 2024 Dec 10;18(1):wrae239. doi: 10.1093/ismejo/wrae239 (PMC11697108; doi:10.1093/ismejo/wrae239)
Supplement: Supplementary_Information_revisionok_04-11-24_wrae239 [file supplementary_information_revisionok_04-11-24_wrae239.docx]

Supplementary Information for

Transportome remodeling of a symbiotic microalga inside a planktonic host

Juery C, Auladell A, Füssy Z, Chevalier F, Yee DP, Pelletier E, Corre E, Allen, AE, Richter DJ, Decelle J

Caroline Juery: [caroline.juery@cea.fr](mailto:caroline.juery@cea.fr); carolinejuery@gmail.com

Johan Decelle: [johan.decelle@univ-grenoble-alpes.fr](mailto:johan.decelle@univ-grenoble-alpes.fr)

**This PDF file includes:**

Fig. S1 to S8

Page 7. Complementary method and Fig. S6 and S7 for metatranscriptomic analysis

**Other supplementary materials for this manuscript include the following:**

Table S1. Excel, Data of figure 1

Table S2. Excel, Data of gene expression of *Phaeocystis* in culture

Table S3. Excel, Data of figure 2

Table S4. Excel, Data of figure 4

Table S5. Excel, Data of figure 5

Table S6 Excel, Matrix of percentage of identity of *Phaeocystis* and acantharia genes

Table S7 Excel, Phaeocystis/Acantharia taxonomic identity


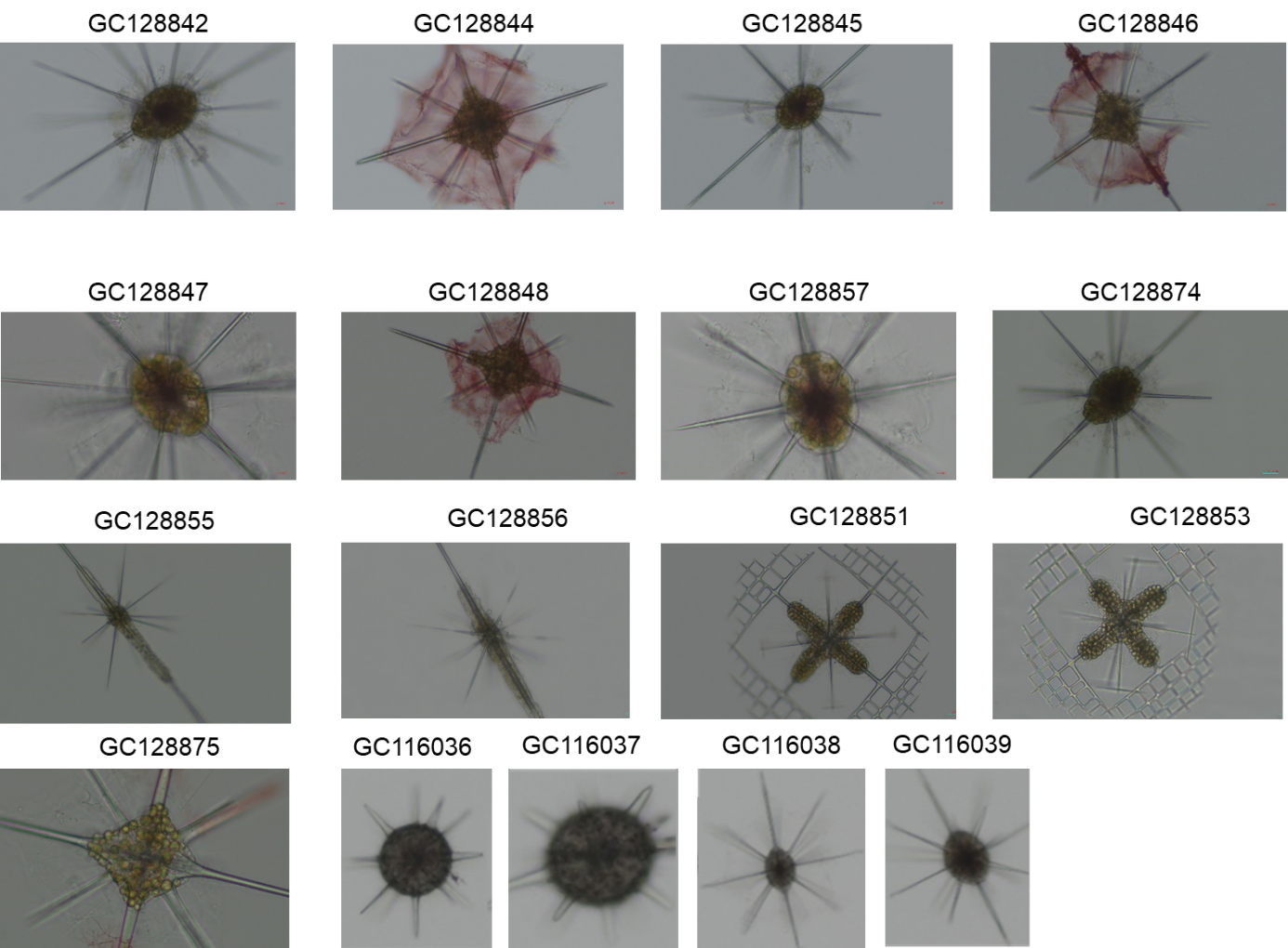


**Figure S1**. Pictures of each samples of symbiotic acantharia used in the DESeq2 analysis.


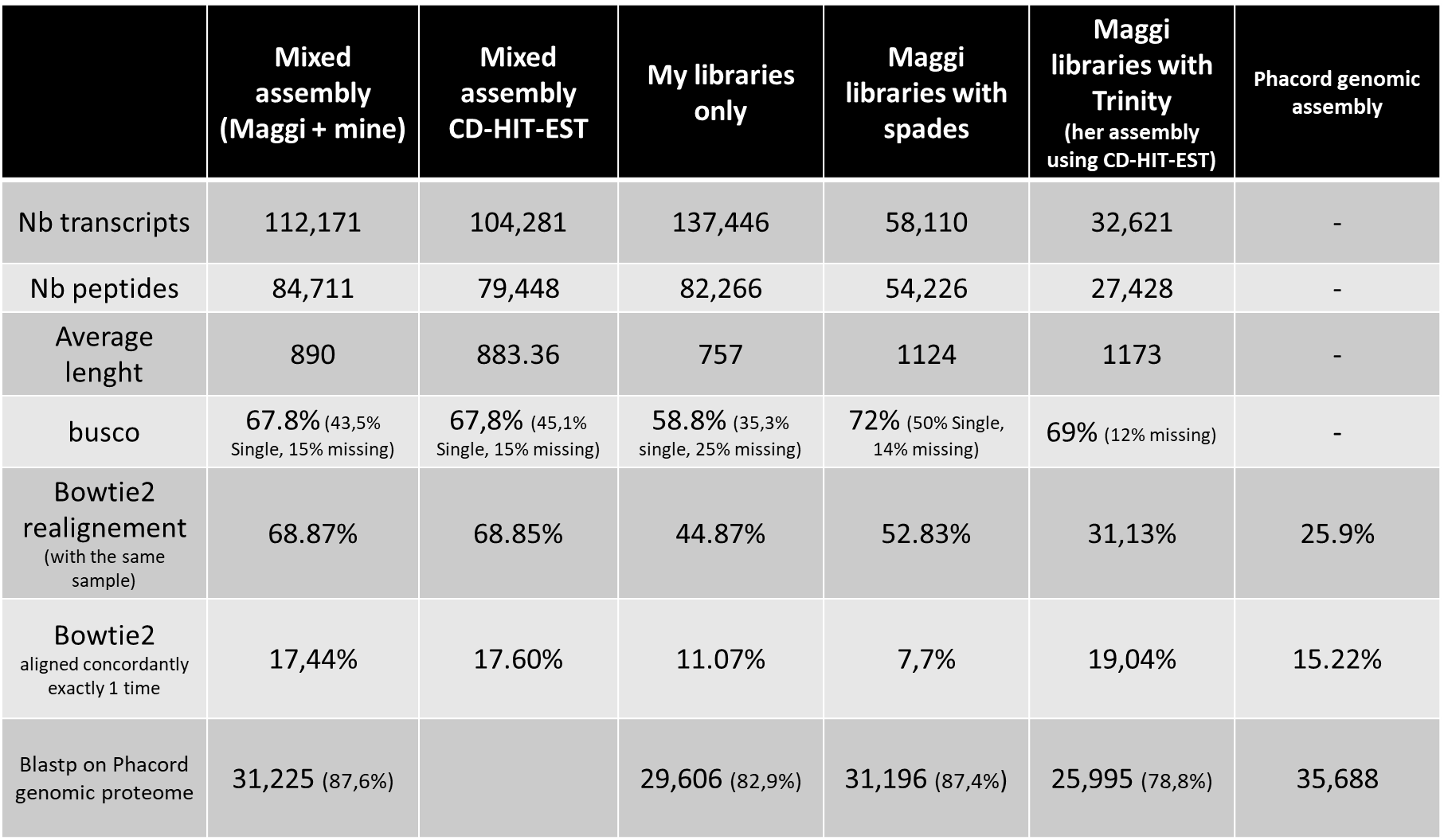

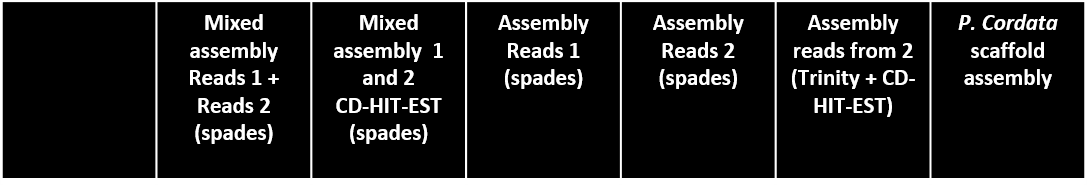


**A**

31,071 (87,1%)


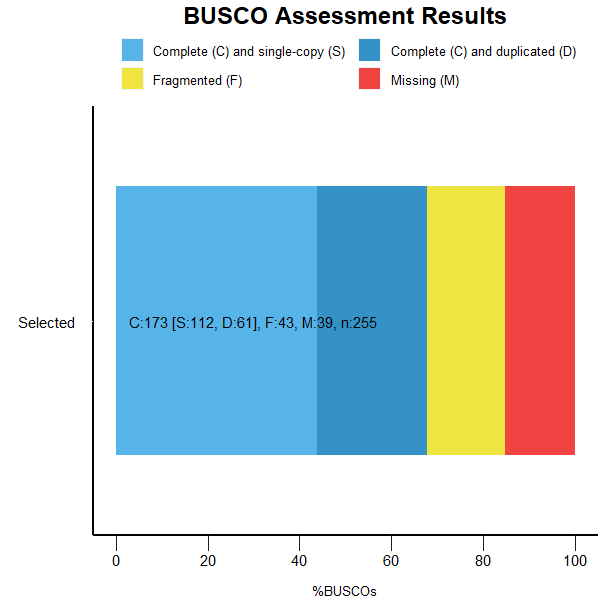


68%

15%

**B**

**Figure S2. Reference transcriptome statistics. A)** Comparison of multiple *Phaeocystis cordata* genome and reference transcriptome assemblies. Reads1 corresponds to the raw reads sequenced in this paper from the nine replicates of the culture of *Phaeocystis cordata;* Reads2 corresponds to the raw reads from PRJNA603434; *P. cordata* scaffold assemblycorresponds to the JGI genomic reference. Two different tools were used to assemble reads for transcriptomes: TrinityV2.1.1 or spadesV3.15.5. Bowtie2 re-alignment corresponds to the mapping rates of the reads re-alignment that were used for the assembly of the transcriptome references.

**B)** BUSCO score of the chosen reference transcriptome from A (Eukaryotic lineage).


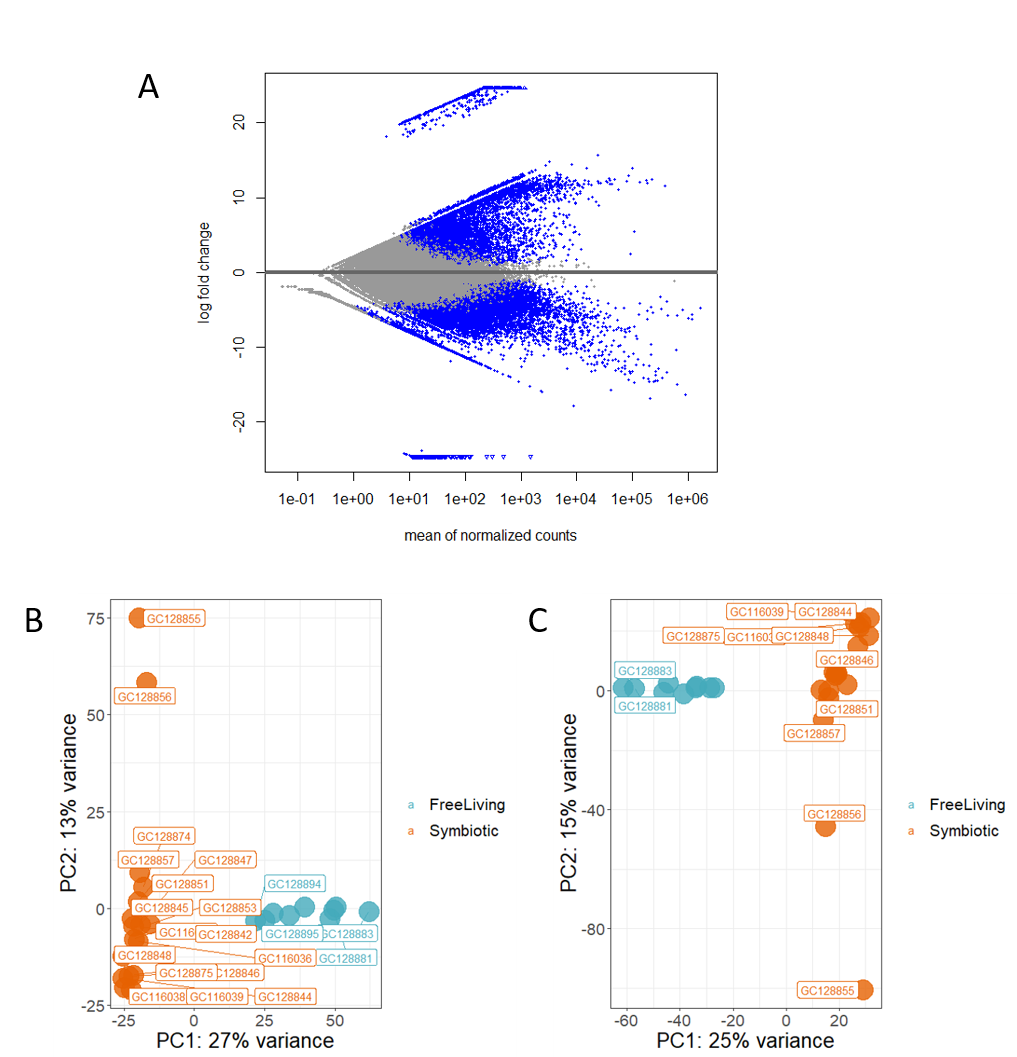


**Figure S3. Comparison of *Phaeocystis cordata* gene expression variance between Free-living and symbiotic samples**. **A**) MA plot of the normalized counts (DESeq2 + preprocessCore R package for quantiles normalization). **B)** and **C)** PCA of gene expression variance between conditions (x-axis) and between replicates (y-axis) for **B)** 1519 kinase genes and **C)** 2817 randomly selected genes.

**
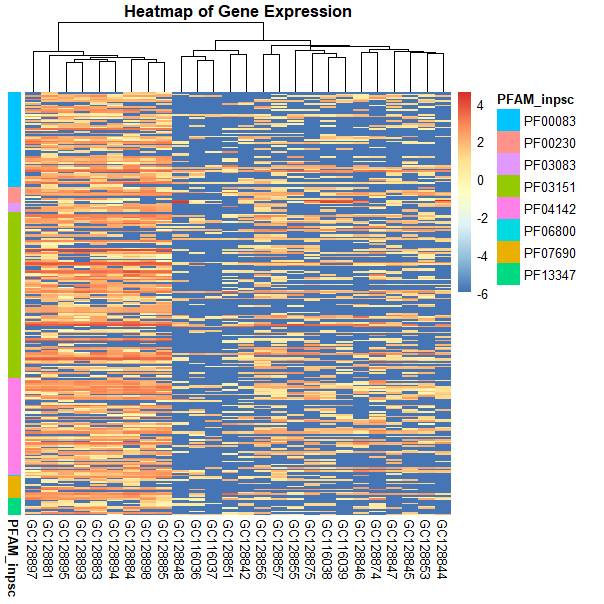
**

**Figure S4.** Heatmap of sugar transporters normalized read counts of free-living and symbiotic samples of *Phaeocystis* used for the DESeq2 analysis. The genes were ordered according to the Pfam they belong to (left).


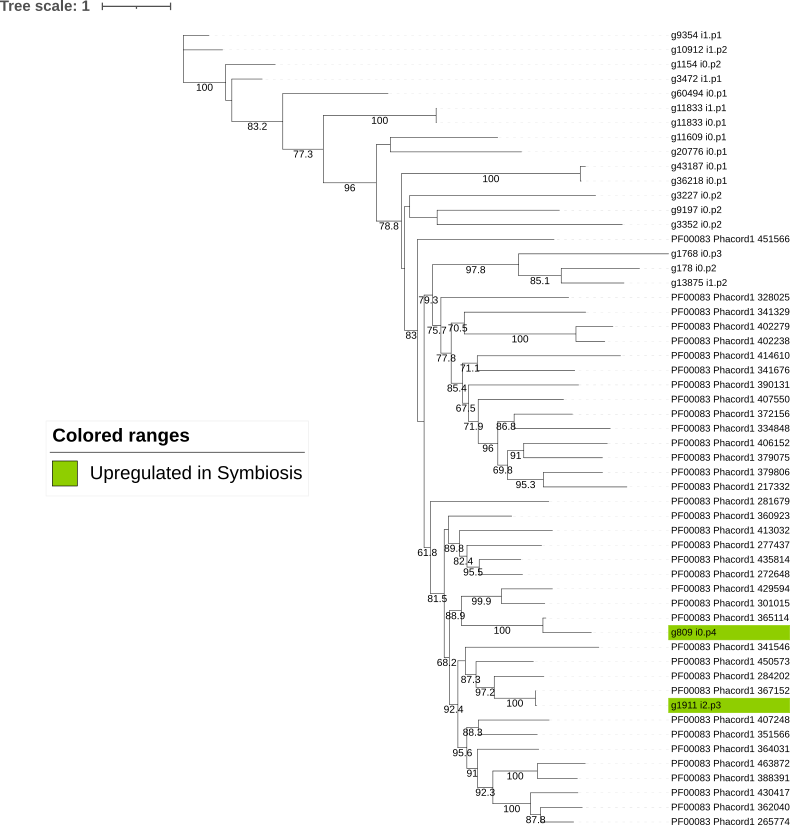


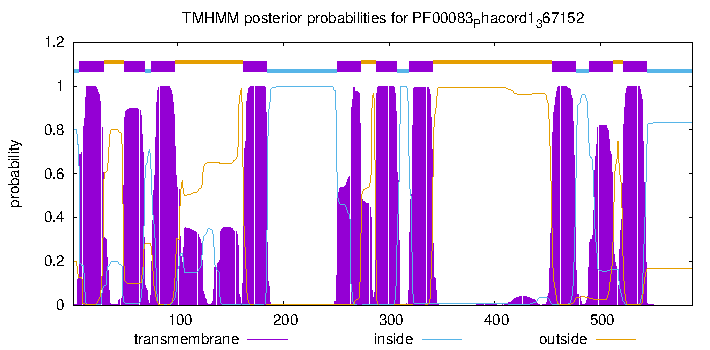

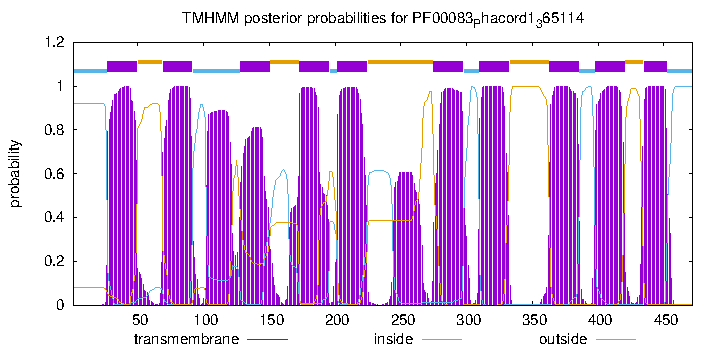


**Figure S5. A) Gene tree of PF00083 *Phaeocystis cordata* protein sequences from JGI genome assembly and the predicted peptides from the reference transcriptome for the two upregulated PF00083 genes (reference transcriptome) in symbiosis.**

Model of substitution: Q.pfam+F+I+G4; 38 sequences with 1681 amino-acid sites; Number of constant sites: 575 (= 34.2058% of all sites); Number of invariant (constant or ambiguous constant) sites: 575 (= 34.2058% of all sites); Number of parsimony informative sites: 616. Number of distinct site patterns: 1329

**B) Transmembrane domain prediction of the two PF00083 predicted peptides upregulated in symbiosis** (TMHMM 2.0).

**Complementary method section for the metatranscriptomic analysis of Figure 4.**

To prevent the risk of reads cross-mapping if there are multiple species of *Phaeocystis* in the Tara samples that could influence our results, we complemented the Salmon mapping with a pairwise comparison with Diamond. The Diamond approach allows us to see the identity of the match, filtering for only closely related matches (>99% identity) and establishing their correspondence to the Salmon results.

When the values are compared, in most cases the correlation is linear as expected, with a slight overinflation towards the Salmon counts, but without relevant changes in the conclusions (Figure S6 below). The only case not presenting a strong correlation is that of g3107_i0.p1, a triose phosphate transporter. For this gene, we observe a higher count in Diamond, deviating from the 1 to 1 correspondence. To understand this difference, we evaluated the distribution of reads over the length of the gene (Figure 7 below). We observed a higher density between the nucleotides 180 and 220 and ending around nucleotides 250. In this region of the gene there is a TMHMM conserved helix domain, which could recruit reads from other species. Through the filtering algorithm of Salmon, based on a uniform distribution over the gene length, most of these mappings are discarded. Overall, these results show that the Salmon mappings are specific to the studied *Phaceocystis* genome.


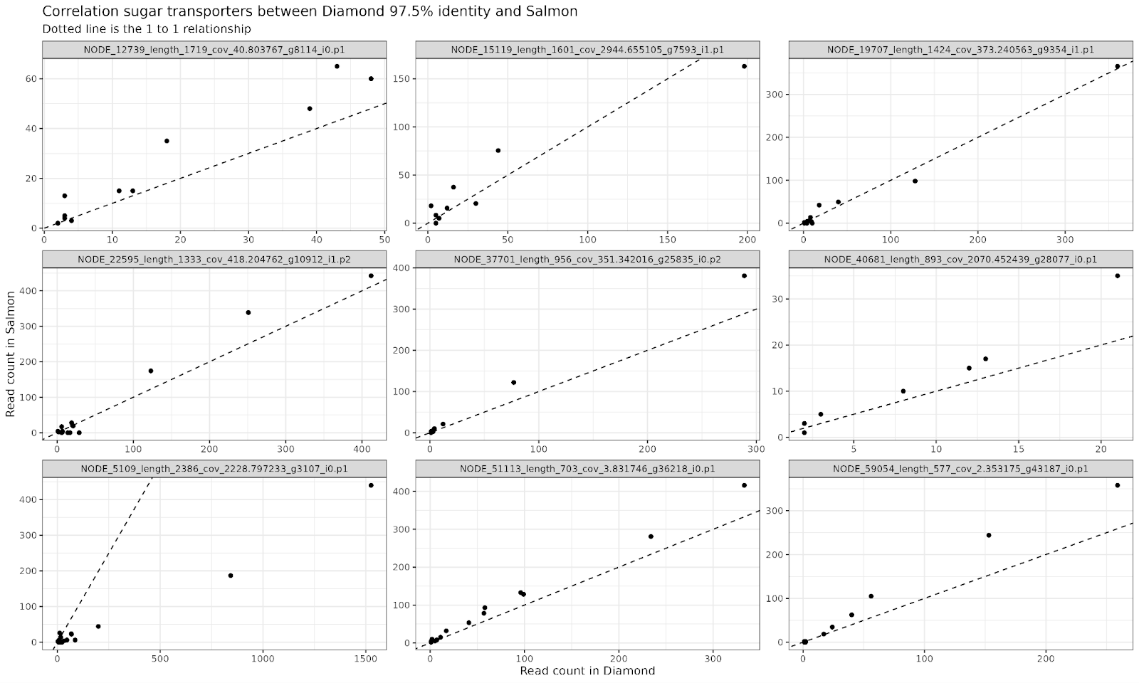


**Figure S6**: Correlation between Salmon and Diamond mappings for the multiple sugar transporters.


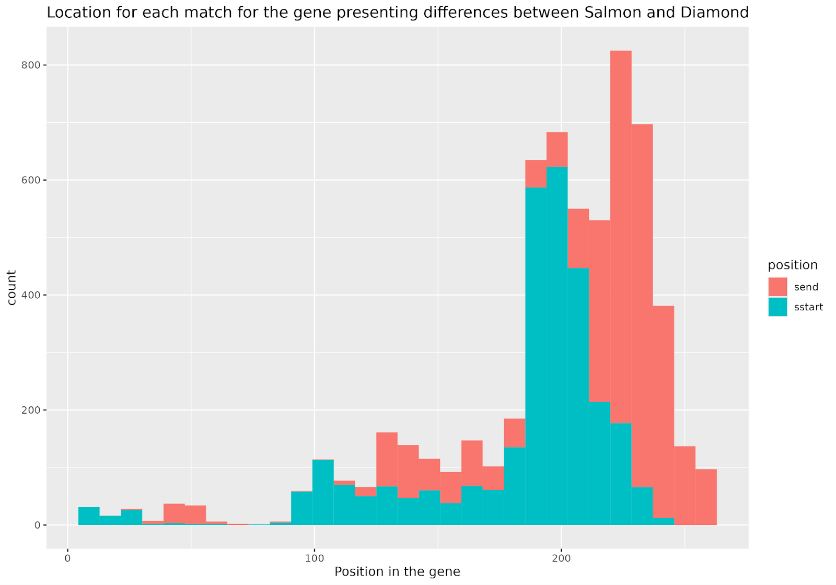


**Figure S7**: Distribution of reads alignment along the gene body of the triose phosphate transporter gene. *Sstart* indicates the location of the start of the read, and *send* the ending.


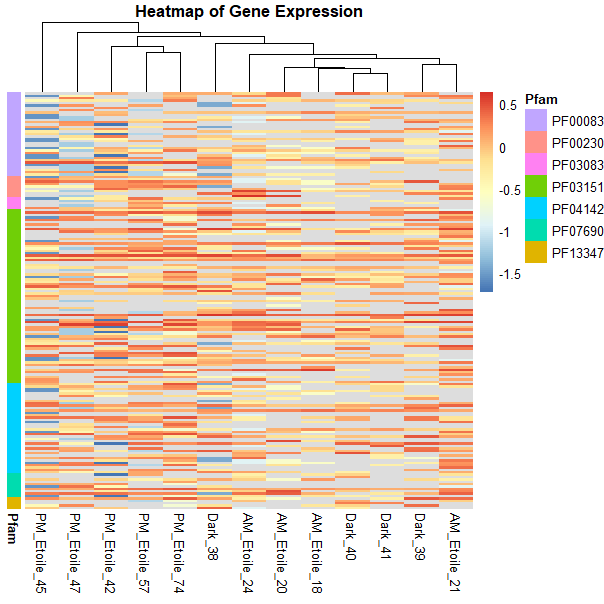


**Figure S8.** Heatmap of symbiotic *Phaeocystis* cordata normalized reads counts (DESeq2 normalization without conditions defining the dds object) of the three different light conditions samples (AM= Morning, PM= Evening and Dark = Dark-evening. The genes were ordered according to the Pfam they belong to (left).
